# Combined treatment with FK506 and nerve growth factor for spinal cord injury in rats

**DOI:** 10.3892/etm.2013.1254

**Published:** 2013-08-07

**Authors:** GUANG CHEN, ZHEN ZHANG, SHOUYU WANG, DECHENG LV

**Affiliations:** Department of Orthopedic Surgery, First Affiliated Hospital, Dalian Medical University, Dalian, Liaoning 116011, P.R. China

**Keywords:** FK506, nerve growth factor, spinal cord injury, neural regeneration

## Abstract

Following spinal cord injury in rats, FK506 is able to protect local nerve tissue, promote neural regeneration, reduce neuronal apoptosis and accelerate the recovery of spinal cord functions. Nerve growth factor (NGF) is important in the regulation of central and peripheral nerve cell regeneration, growth differentiation and functions. Previous studies have shown that FK506 and NGF exhibit a synergistic effect in the treatment of peripheral nerve injury; however, it remains unclear whether the synergistic effect is present in the treatment of spinal cord injury. In this study, we combined FK506 and NGF for the treatment of spinal cord injury in rats. The NF200 protein expression in rats with spinal cord injury was determined using immunohistochemical staining and NF200 mRNA expression levels were observed using the reverse transcription-polymerase chain reaction method. The restoration of spinal cord functions was evaluated using the Basso, Beattie and Bresnahan score. The results demonstrated that the combined treatment significantly enhanced the expression of NF200 and improved spinal cord functions compared with the results of the single treatment. Our experimental observations indicated that FK506 and NGF exhibit a synergistic effect in the treatment of spinal cord injury in rats and that the combined treatment may effectively promote neural regeneration and functional recovery in rats following spinal cord injury.

## Introduction

FK506 (tacrolimus) is a type of macrolide immunosuppressant drug that has been widely used in organ transplantation. In addition to exhibiting an immunosuppressive effect with a high efficiency and low toxicity, FK506 also promotes neural regeneration. Gold *et al* (1) first reported the application of FK506 in the treatment of rats with sciatic nerve crush injury and observed that FK506 accelerated the injured nerve regeneration and promoted the recovery of neural function (1,2). A number of studies investigating the role of FK506 in different models of spinal cord injury have also demonstrated the neurotrophic and neuroprotective effects of FK506 and its contribution to functional recovery following spinal cord injury (3–5), thereby indicating a potential novel pathway for drug therapy of spinal cord injury. Nerve growth factor (NGF) is an important member of the neurotrophic factor family, which is widely located in peripheral tissues, the peripheral and central nervous systems and is a critical factor in neuronal development and survival, axonal remodeling and function, as well as in the repair process following spinal cord injury (6,7). When spinal cord injury occurs, NGF is expressed in the injured tissues, which has a positive effect on the prevention of secondary injury caused by microenvironmental changes. However, the expression level is low and, therefore, exogenous NGF may be considered as a means of treating spinal cord injury. At present, *in vivo* and *in vitro* studies have demonstrated the synergistic effect of FK506 and NGF in the treatment of peripheral nerve injuries (8–11); however, it remains unclear whether this effect is present in the treatment of spinal cord injury. The aim of this study was to observe whether FK506 and NGF exhibited a synergistic effect on the recovery of spinal cord functions following acute spinal cord injury in rats.

## Materials and methods

### Materials

A total of 120 Sprague-Dawley female clean rats, weighing 180–220 g, were provided by the Animal Experimental Center of Dalian Medical University, China [license No. SCXK (Liao) 2008–0002]. The study was approved by the Animal Research Ethics Committee of Dalian Medical University. All experimental procedures were in accordance with the Guidance Suggestions for the Care and Use of Laboratory Animals, published by the Ministry of Science and Technology of the People’s Republic of China (2006-09-30).

### Grouping and establishment of models

The 120 female rats were randomly divided into five groups: control, FK506 treatment, NGF treatment, FK506 plus NGF treatment and sham surgery, with 24 rats in each group. After being weighed, the rats were anesthetized with 10% chloral hydrate (300 mg/kg) via intraperitoneal injection and fixed in the prone position. Following this, a dorsal midline incision was made under sterile conditions and models of spinal cord injury were established using the modified Allen’s method (12). In brief, T9–T10 spinous processes and lamina were excised, exposing the spinal dura mater. A 5-g iron hammer was then allowed to fall freely from a 5-cm height on to the dural sac, at a strength of 5 g × 5 cm and a damage diameter of 2 mm. The rats exhibited tail flicking immediately following the attack, retraction of the hind limbs and the body and then hind limb paralysis. These manifestations indicated the success of the modeling. In the sham surgery group, only the T9–T10 segment laminectomy was performed, with no attack on the spinal cord. At 30 min subsequent to injury, the rats in the three treatment groups were treated with 0.3 mg/kg FK506 (Sigma-Aldrich, St. Louis, MO, USA), 40 *μ*g/ kg NGF (Staidson Biopharmaceuticals Co., Ltd, Beijing, China) and 0.3 mg/kg FK506 plus 40 *μ*g/kg NGF, respectively, via intraperitoneal injection, once a day, for one week.

### Specimens from the injury area

Three rats were randomly selected from each group at each time-point and the NF200 protein expression was determined using immunohistochemical methods. Rats were anesthetized with 10% chloral hydrate via intraperitoneal injection, the chest was opened and rats were fixed in 4% paraformaldehyde for cardiac perfusion until body stiffness was present. This took 20–30 min. Following the completion of the perfusion, the injured spinal cord tissues were harvested and 1.0-cm-long specimens were stored in 4% paraformaldehyde for 24 h and embedded in paraffin.

A further three rats were randomly selected from each group at each-time point for the detection of NF200 mRNA expression, using the reverse transcription-polymerase chain reaction method. Rats were anesthetized with 10% chloral hydrate via intraperitoneal injection, prior to the injured spinal cord tissues being harvested (∼100 mg). The specimens were stored at −80°C.

### Immunohistochemical detection of NF200 protein expression

Paraffin specimens of 4-*μ*m thickness were hydrated and rinsed with phosphate-buffered saline (PBS; pH 7.4) three times, for 3 min each, prior to being incubated with 3% hydrogen peroxide solution at room temperature for 10 min and rinsed with PBS a further three times for 3 min each. Specimens were then microwave repaired with 0.01 M carbonate buffer (CB) (pH 6.0) at 100°C for 15 min and naturally cooled to room temperature, prior to being rinsed with PBS three times for 3 min each. Specimens were blocked with 5% goat serum at room temperature for 10 min and were subsequently incubated with NF200 antibody (1:100) at 4°C overnight and rinsed with PBS three times for 3 min each. Each section was then incubated with goat anti-rabbit antibody at 37°C for 30 min and rinsed with PBS three times for 3 min each. Following this, 3,3’-diaminobenzidine (DAB) coloration was performed for 10 min and the specimens were rinsed with tap water for 10 min, prior to being counterstained with hematoxylin for 10 sec, dehydrated and mounted.

Five visual fields randomly selected from the injured spinal cord sections were observed using the Image-Pro Plus 6.0 medical image analysis system (Media Cybernetics, Inc., Silver Spring, MD, USA) and the average intracellular optical density (OD) value for the NF200 staining was calculated.

### Reverse transcription-polymerase chain reaction detection of NF200 mRNA expression

Total RNA extraction from the specimens was performed according to the instructions of the RNAiso Plus kit [Takara Biotechnology (Dalian) Co., Ltd., Dalian, China] and the reverse transcription-polymerase chain reaction primers were designed using Primer 5.0 software (NF200: 5’-GCA GAC ATT GCC TAC C-3’ and 5’-TCA CTC CTT CCG TCA CCC-3’; and β-actin: 5’-GTA AAG ACC TCT ATG CCA ACA-3’ and 5’-CCT TCA CCG TTC CAG TTT-3’, forward and reverse, respectively). The polymerase chain reaction was performed according to the instructions of the PrimeScript® One Step RT-PCR kit version 2 [Dye Plus; TaKaRa Biotechnology (Dalian) Co., Ltd]. In brief, the cycle conditions for NF200 were: 50°C for 30 min and 94°C for 2 min, followed by 30 cycles at 94°C for 30 sec, 57°C for 30 sec and at 72°C for 1 min. The conditions for β-actin were: 50°C for 30 min and 94°C for 2 min, followed by 30 cycles at 94°C for 30 sec, 52°C for 30 sec and at 72°C for 1 min. Polymerase chain reaction products were detected with 2% agarose gel electrophoresis and analyzed with a gel imaging analyzer. The ratio of the target gene (NF200) OD to the reference gene (β-actin) OD in the same specimen was calculated and considered as the target gene mRNA relative content.

### Assessment of spinal motor function

The spinal cord functions were assessed by the Basso, Beattie and Bresnahan (BBB) scale (13). Five rats from each group were randomly selected at 3, 7, 14 and 21 days post-injury and their lower limb functions were tested using BBB scores. Hind limb paralysis was scored as 0 points, while a completely normal spinal cord was scored as 21 points. The spinal cord functions were scored according to the number and motion range of the joints as well as limb and tail activities. The assessment was performed by two physicians independently within 5 min, using a double-blind method, and the average values of the two test results were taken as the recording values.

### Statistical analysis

Statistical analysis was performed using SPSS 17.0 statistical software (SPSS, Inc., Chicago, IL, USA) and data are expressed as the mean ± standard deviation. Multiple groups were compared using one-way analysis of variance and differences between two groups were compared using the Student’s t-test. P<0.05 was considered to indicate a statistically significant difference.

## Results

### NF200 protein expression following spinal cord injury

**Figure 1.**
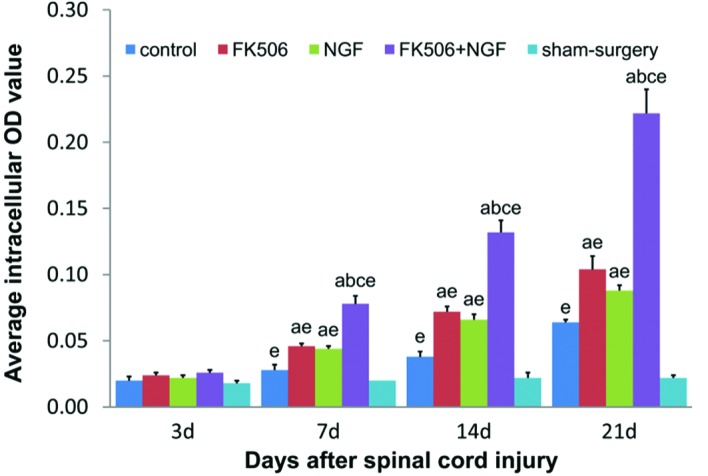
Average intracellular optical density (OD) values of NF200 staining in each group at each time-point. ^a^P<0.05, vs. the control group at the same time-point; ^b^P<0.05, vs. the FK506 group at the same time-point; ^c^P<0.05, vs. the nerve growth factor (NGF) group at the same time-point; ^d^P<0.05, vs. the FK506 + NGF group at the same time-point; ^e^P<0.05, vs. the sham surgery group at the same time-point.

**Figure 2.**
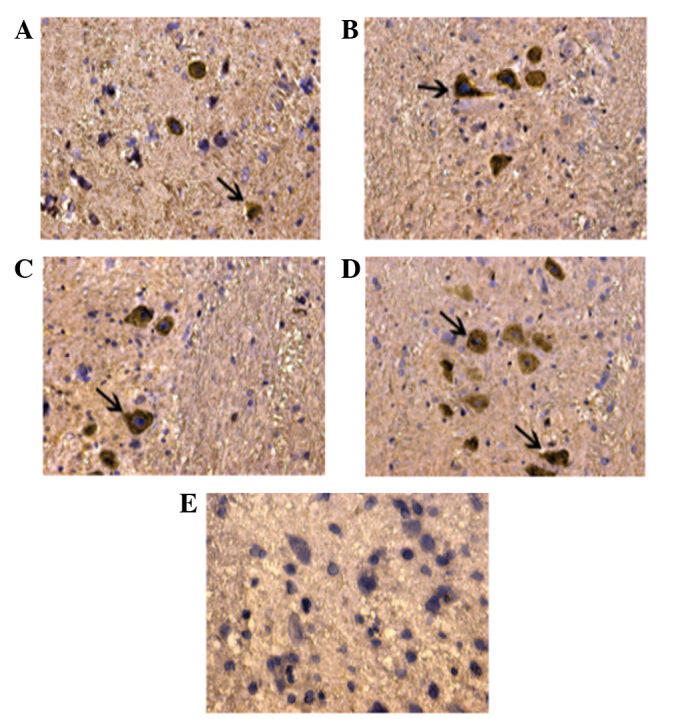
NF200 staining at 21 days subsequent to injury in each group (immunohistochemistry; magnification, ×200). NF200 immunohistochemical staining in the (A) control, (B) FK506, (C) nerve growth factor (NGF), (D) FK506 + NGF and (E) sham surgery groups. Arrows indicate the NF200 positive cells.

The NF200 expression is shown in Figs. 1 and 2. There were no significant differences in the average intracellular NF200 staining OD values among the groups 3 days after the injury (P>0.05). At 7, 14 and 21 days post-injury, the average intracellular NF200 staining OD values were shown to have gradually increased in all the treatment groups and the control group, with the treatment groups showing significantly higher expression levels than the control and sham surgery groups (P<0.05). In the FK506 plus NGF treatment group, the average intracellular NF200 staining OD value was significantly higher than the ODs in the FK506 alone and NGF alone groups (P<0.05), with no significant difference between the FK506 and NGF groups.

### Detection of cells positive for NF200 staining

There were few cells positive for NF200 staining in the control group. However, there were significantly increased numbers of cells positive for NF200 staining in all the treatment groups, with a higher number in the FK506 plus NGF treatment group than in the groups treated with FK506 or NGF alone.

**Figure 3.**
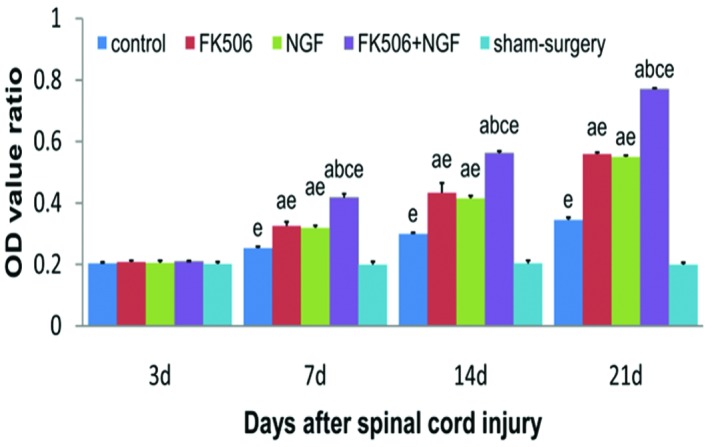
Histogram of the NF200 mRNA expression levels in each group at each time-point. ^a^P<0.05, vs. the control group at the same time-point; ^b^P<0.05, vs. the FK506 group at the same time-point; ^c^P<0.05, vs. the nerve growth factor (NGF) group at the same time-point; ^d^P<0.05, vs. the FK506 + NGF group at the same time-point; ^e^P<0.05, vs. the sham surgery group at the same time-point.

**Figure 4.**
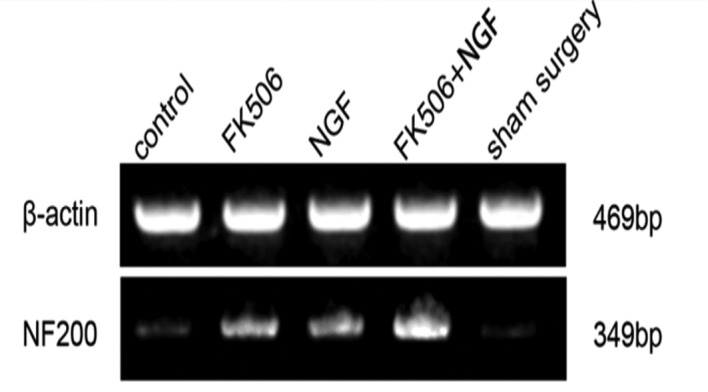
Reverse transcription-polymerase chain reaction detection of NF200 mRNA expression at 21 days subsequent to injury. β-actin was used as a loading control. NGF, nerve growth factor.

The reverse transcription-polymerase chain reaction was performed to further confirm the results of the immunohisto-chemistry (Figs. 3 and 4). There were no significant differences in the OD value ratios of NF200 mRNA at 3 days after injury (P>0.05). At 7, 14 and 21 days post-injury, the OD value ratios of NF200 mRNA were shown to have gradually increased in all treatment groups and the control group, with the treatment groups showing significantly higher expression levels than the control and sham surgery groups (P<0.05). In the FK506 plus NGF treatment group, the OD value ratio of NF200 mRNA was significantly higher than those in groups treated with FK506 or NGF alone (P<0.05), with no significant difference between the FK506 and NGF groups.

### Lower limb function recovery following spinal cord injury

**Figure 5.**
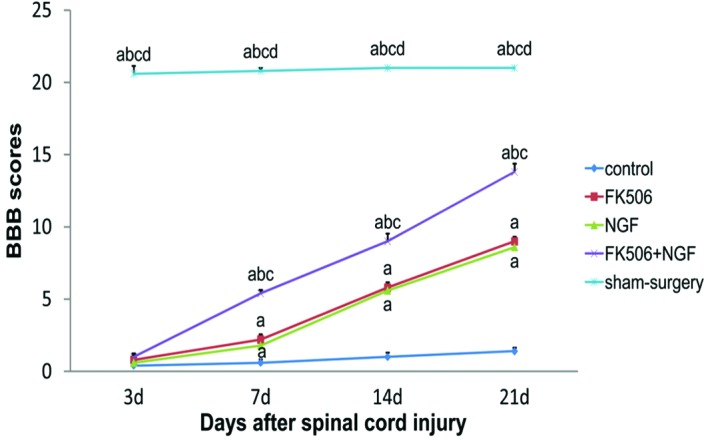
Comparison of Basso, Beattie and Bresnahan BBB scores. ^a^P<0.05, vs. the control group at the same time-point; ^b^P<0.05, vs. the FK506 group at the same time-point; ^c^P<0.05, vs. the nerve growth factor (NGF) group at the same time-point; ^d^P<0.05, vs. the FK506 + NGF group at the same time-point; ^e^P<0.05, vs. the sham surgery group at the same time-point.

The BBB scores at each time-point are shown in Fig. 5. There were no significant differences in the BBB scores among the groups, with the exception of the sham surgery group, at 3 days subsequent to injury. Furthermore, the BBB scores in the treatment groups were significantly higher than those in the control group at 7, 14 and 21 days post-injury (P<0.05), and were significantly increased in the FK506 plus NGF treatment group compared with those in the groups treated with FK506 or NGF alone (P<0.05). There was no significant difference in the BBB scores between the FK506 and NGF groups (P>0.05).

## Discussion

Neurofilament protein NF200 is the main component of the neuronal and axonal cytoskeleton and is of particular significant in the maintenance of neuronal functions, axoplasmic transport and a series of pathophysiological changes associated with the repair process following spinal cord injury (14). In normal circumstances, although NF200 exists in neurites, it is scarcely seen in cell bodies; therefore, the normal NF200 staining result is negative. Following spinal cord injury, cells at the area of the injury degenerate and become necrotic, while the adjacent neurons may synthesize a large quantity of NF200 under the stimulation of the insult. Accordingly, these adjacent neurons may show cell body staining (15). A previous study observed a close correlation between the number of NF200-positive neurons/the degree of neuronal cell body staining and lower limb functional recovery following incomplete spinal cord injury (16). The aim of the current study was to observe NF200 staining, in a broader attempt to investigate the morphology and functions of neurons following injury.

NGF is a type of polypeptide growth factor that exerts biological effects on the development, repair and regeneration of the central nervous system through selective binding with the high affinity receptor, TrkA (10,17,18), and is very important in the repair process following spinal cord injury (19). In a previous study (20), the NGF receptor mRNA levels were shown to be significantly increased at day 4 subsequent to spinal cord injury and were observed to peak at day 7, at levels five-seven-sfold as high as those in the control group. Even 14–28 days subsequent to injury, the level remained four-fold that of the control group. In the current study, the BBB scores and NF200 protein and mRNA expression levels in the NGF treatment group were higher than those of the control group at 7, 14 and 21 days post-injury, with statistically significant differences (P<0.05). These experimental observations were consistent with those regarding the NGF receptor expression; therefore, it was suggested that exogenous NGF may promote neural regeneration and functional recovery in rats with spinal cord injury, with 7 days post-injury as the optimal treatment-point. The drug delivery method in the present study was intraperitoneal injection; although this was convenient, the biological utilization rate was low. Therefore, the requirement for a synergistic drug for NGF treatment is increasing in the field of spinal cord injury.

FK506 serves as an immunosuppressive agent and also promotes neural regeneration (21,22). A number of studies (23,24) have shown that FK506 upregulates the expression of growth associated protein (GAP)-43 in neuronal cells by inhibiting the CaN activity and promotes neurite extension and neuronal recovery following spinal cord injury. In addition FK506 has been demonstrated to suppress cysteine proteinase-3 activation in oligodendrocytes and reduce neuronal apoptosis following spinal cord injury (25,26). With regard to the correlation between FK506 and NGF, Lyons *et al* (8) observed that NGF upregulated the FK506 binding protein levels in PC12 cells, while FK506 enhanced the PC12 cell sensitivity to NGF and reduced the NGF concentration by 20-50-fold. Jifeng *et al* (11) applied the combined treatment of an FK506 and NGF composite membrane to repair the injured sciatic nerve in rats and showed that the combined treatment was more effective than single applications and was able to reduce the dosage of the immunosuppressant, FK506. In a study by Price *et al* (10), FK506 was shown to promote NGF expression and induce axonal outgrowth. Furthermore, Gold *et al* (27) demonstrated that, whereas the immunosuppressive effect of FK506 was dependent on the immunophilin FKBP12, the FK506 pro-nerve growth effect was mediated by binding to the immunophilin FKBP-52, through the FKBP52/HSP90/steroid receptor (SR) complex (27). FK506 binding with FKBP-52 was shown to separate heat shock protein 90 and FKBP52 from the complexes, with heat shock protein 90 acting with mitogen-activated protein kinase/extracellular signal-regulated kinase 2 (28), in a pathway that may be cross-linked to NGF signaling pathways (29,30). This is indicative of the synergistic mechanism.

In the current study, animal models of acute spinal cord injury were established using the modified Allen’s method and the successful models were treated with FK506 and/or NGF intraperitoneal injection 30 min after modeling, once a day for seven days. At day 7 subsequent to injury, the BBB scores and NF200 expression in all treatment groups were significantly higher than those in the control group, and the BBB scores and NF200 expression of the combined treatment group were higher than those of the single treatment group. Our experimental results showed the synergistic effects of FK506 and NGF in the treatment of spinal cord injury, and demonstrated that the combined treatment was able to effectively promote neural regeneration and functional recovery in rats following spinal cord injury. We consider that combined treatment with FK506 and NGF is able to increase the biological utilization efficiency of FK506 and exogenous NGF in the treatment of spinal cord injury and that the neurotrophic and neuroprotective mechanisms of the combined treatment exhibited a synergistic effect on NF200 expression, effectively promoting neural regeneration and functional recovery following spinal cord injury. This may provide a new treatment means for acute spinal cord injury.
